# Effect of pinolenic acid on oxidative stress injury in HepG2 cells induced by H_2_O_2_

**DOI:** 10.1002/fsn3.2534

**Published:** 2021-08-25

**Authors:** Yang Zhao, Sainan Liu, Zhili Sheng, Xue Li, Yanan Chang, Weichang Dai, Sui Kiat Chang, Junmei Liu, Yuchun Yang

**Affiliations:** ^1^ College of Food Science and Engineering Jilin Agricultural University Changchun China; ^2^ Key Laboratory of South China Agricultural Plant Molecular Analysis and Genetic Improvement Key Laboratory of Post‐Harvest Handling of Fruits Ministry of Agriculture South China Botanical Garden Chinese Academy of Sciences Guangzhou China; ^3^ Forestry Academy of Jilin Province Changchun China

**Keywords:** antioxidant defense responses, antioxidant enzymes system, HepG2 cells, oxidative stress, Pinolenic acid

## Abstract

To investigate the effect and mechanism of pinolenic acid (PNA) on H_2_O_2_‐induced oxidative stress injury in HepG2 cells. Methods: PNA was used to regulate oxidative stress injury of HepG2 cells induced by H_2_O_2_. Quantification of cell survival rate, accumulation of intracellular reactive oxygen species (ROS), and expression levels of anti‐oxidation‐related genes were determined using MTT, fluorescent probe technology (DCFH‐DA), and real‐time quantitative reverse transcription polymerase chain technology (qRT‐PCR) method, respectively. Meanwhile, the activity of intracellular antioxidant enzymes was determined by biochemical methods. The results showed that PNA improved the survival rate of HepG2 cells induced by H_2_O_2_ (29.59%, high‐dose group), reduced the accumulation of intracellular ROS (65.52%, high‐dose group), and reduced the level of intracellular malondialdehyde (MDA; 65.52%, high‐dose group). All these results were dose‐dependent, which indicated that PNA can improve oxidative stress damage of cells. Furthermore, the mechanism of PNA regulating oxidative stress was investigated from the gene level. Results showed that under supplementation of PNA, the activity of superoxide dismutase (SOD), catalase (CAT), and glutathione peroxidase (GSH‐Px) had been improved (39.74%, 17.58%, and 23.83%, high‐dose group). Further studies on gene expression which controls the activity of antioxidant enzymes showed that under the regulation of PNA, the expression level of Keap1 gene was decreased, while Nrf2 gene was increased. The expression levels of HO‐1 and NQO1 in the downstream of Nrf2 were increased. Results indicated that under the regulation of PNA, Nrf2 was separated from Keap1, entered the nucleus, bound to ARE, and up‐regulated the expression levels of HO‐1 and NQO1 genes. Conclusion: PNA has a conspicuous improvement effect on oxidative stress damage induced by H_2_O_2_ in HepG2 cells. We also found the antioxidant mechanisms of PNA where it protected cells from oxidative stress damage by causing nuclear translocation of Nrf2 gene and up‐regulated the expression levels of antioxidant enzymes in the downstream. This shows that PNA prevented oxidative stress by mediating the Keap1/Nrf2 transcriptional pathway and down‐regulating enzyme activities.

## Graphical abstract

Oxidative stress injury of HepG2 cells induced by H_2_O_2_ was regulated by Pinolenic acid. The antioxidant ability of the body was strengthened by Pinolenic acid; thus, the ability to regulate oxidative stress from outside was enhanced. The cellular oxidative stress was regulated by Pinolenic acid, which was dose‐dependent, and the higher the concentration, and the better the regulation ability.

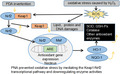

## 1 INTRODUCTION

Excessive reactive oxygen species (ROS) and other free radicals cause oxidative damage which further contribute to numerous diseases, such as aging, cancer, and neurodegenerative diseases, such as dementia and Alzheimer's disease (Wang, 2015). Hence, decreasing oxidative damage may help to prevent those diseases. Oxidative damage constantly occurs in vivo where damaged biomolecules must be repaired. Failure to replace the damaged biomolecules enhances oxidative damage and contributes to inflammation (Long et al., 2020; Yueming et al., 2020). Dietary antioxidants from natural sources play important roles by reducing oxidative damage that may help to reduce the severity of chronic diseases, as well as to extend the shelf life of food products (Chang et al., 2021; Poljsak & Milisav, 2012). Research on natural plant antioxidants has always been promising since it generates findings for practical applications in the field of human nutrition. Natural antioxidants have shown promising anti‐inflammatory, anti‐bacteria, skin‐whitening, as well as disease preventive properties (Lourenço et al., 2019). PUFAs have been shown to function as antioxidants at the plasma membranes by regulating the antioxidant signaling pathways (Oppedisano et al., 2020). Supplementation with omega‐3 PUFAs improved the antioxidant defense by enhancing the actions of antioxidant enzymes, such as superoxide dismutase (SOD) and glutathione peroxidase (GSH‐Px) (Rodrigo et al., 2013).

PNA (all cis‐5, 9, 12‐18:3) is a unique polyunsaturated fatty acid. Its chemical formula is C_18_H_30_O_2,_ and it is the isomer of linolenic acid (Xie et al., 2016). PNA is the major Δ5‐unsaturated polymethylene‐interrupted fatty acid (Δ5‐UPIFA) in pine nuts and their oil, which accounts for about 15% of the total fatty acids, and it can be as high as 17%–20% in red pine seed oil (No et al., 2015). The chemical structure of PNA is similar to γ‐linolenic acid (GLA) and α‐linolenic acid (ALA). GLA is an n‐6 PUFA while ALA is an n‐3 PUFA, and it is an isomer of GLA (Ryan et al., 2006). Pine nuts and pine nuts oil have been regarded as functional food in China, Korea, and Japan for many years (Zhang et al., 2019). Numerous studies demonstrated that PNA has various health benefits such as antioxidant, weight loss, lipid‐lowering, anti‐inflammation, appetite control, improving insulin sensitivity, cardio‐protection, and anti‐cancer (Le et al., 2012; Xie et al., 2016; Zhang et al., 2019). Previous study demonstrated that PNA has beneficial effect on antioxidant protective mechanisms in rats fed with high‐fat diet (Chen et al., 2011). Recently, Zhang et al. (2019) demonstrated that PNA acts as an antioxidant by alleviating cellular oxidative stress which is beneficial to prevent non‐alcoholic steatohepatitis. However, the antioxidative mechanisms of PNA have not been fully elucidated.

The objective of this study was to determine whether supplementation of PNA alleviates H_2_O_2_‐induced oxidative stress in HepG2 cells. Besides, regulation of ROS production and related antioxidant enzymes defense system by PNA was also evaluated. By detecting the MDA content and intracellular ROS accumulation of HepG2 cells, figure out whether PNA can regulate the oxidative stress response and improve the oxidative stress damage of the cells; by detecting the activity of intracellular antioxidant enzyme system in HepG2 cells and by detecting the expression levels of related antioxidant genes in HepG2 cells, explore the mechanism of which PNA regulates the expression levels of Kelch‐like ECH‐associated protein 1 (Keap1) and Nrf2 genes and study the variation of HO‐1 and NQO1 gene expression levels. Based on the above test results, this experiment may clarify the mechanism of which PNA improves cellular oxidative stress injury and provides a theoretical basis for the development of antioxidant functional foods of PNA and Korean Pine Oil.

## 2 MATERIALS AND METHODS

### 2.1 Materials and reagents

PNA (99.9% purity) was purchased from Sigma‐Aldrich Co. (St. Louis, MO, USA). Human hepatocellular carcinoma (HepG2) cells were purchased from American Type Culture Collection (ATCC; Manassas, VA, USA). Dulbecco's modified Eagle's medium (DMEM), penicillin/streptomycin, phosphate‐buffered saline (PBS) buffer solution, and 3‐(4,5‐Dimethylthiazol‐2‐yl)‐2,5‐diphenyltetrazolium bromide (MTT) were purchased from CASMART, China. Fetal Bovine Serum (FBS), TRIzol reagent, and trypsin were purchased from Gibco (Gibco/BRL Life Technologies, Grand Island, NY, USA). Chloroform, Isopropyl alcohol, and hydrogen peroxide (H_2_O_2_) were supplied by Beijing Chemical Factory Co., Ltd. Bicinchoninic acid (BCA) was obtained from Ding Guo Biotechnology Co., Ltd. Kits used to determine the levels of ROS, malondialdehyde (MDA), superoxide dismutase (SOD), catalase (CAT), and glutathione peroxidase (GSH‐Px) were supplied by Jiancheng Bioengineering Institute (Nanjing, China). PrimeScript™ RT Reagent Kit with gDNA Eraser and TB Green™ Premix Ex TaqTM^II^ was supplied by Takara Biotechnology, Japan. All other reagents used in this study were of analytical grade.

### 2.2 Instruments and equipment

CO_2_ cell incubator, American Thermo; fluorescence inverted microscope, Germany Leica; Microporous plate enzyme marker, Microporous plate luciferase marker, Switzerland Tecan company; desktop high‐speed refrigerated centrifuge, Shanghai Centrifuge Machinery Research Institute; trace nucleic acid protein detector Eppendorf, Germany.

### 2.3 Methods

#### 2.3.1 Cell culture

HepG2 cells were maintained in DMEM with 10% heat‐inactivated FBS (with 1% penicillin–streptomycin) and incubated at 37°C in a humidified atmosphere of 95% air and 5% CO_2_ based on the method of Hao (2018). The growth medium was prepared according to the procedures recommended by the American Tissue Cell Culture (Tang et al., 2021). In all experiments, 80%–90% confluent HepG2 cells were used before treatment. Five treatments were grouped as follows: Cells incubated with serum‐free medium were treated as a control group: Cells incubated with serum‐free medium followed by H_2_O_2_ solution (0.2 mM) for 12 h and then cultured for 24 h were treated as a model group; Cells incubated with medium containing H_2_O_2_ solution (0.2 mM) for 12 h and then incubated with medium containing PNA for 24 h were treated as the experimental groups. The experimental groups were divided into PNA low‐dose (PNA‐L), middle‐dose (PNA‐M), and high‐dose groups (PNA‐H) at 1, 5, and 10 µM, respectively.

#### 2.3.2 Preparation of pinolenic acid (PNA)

**FIGURE 1:**
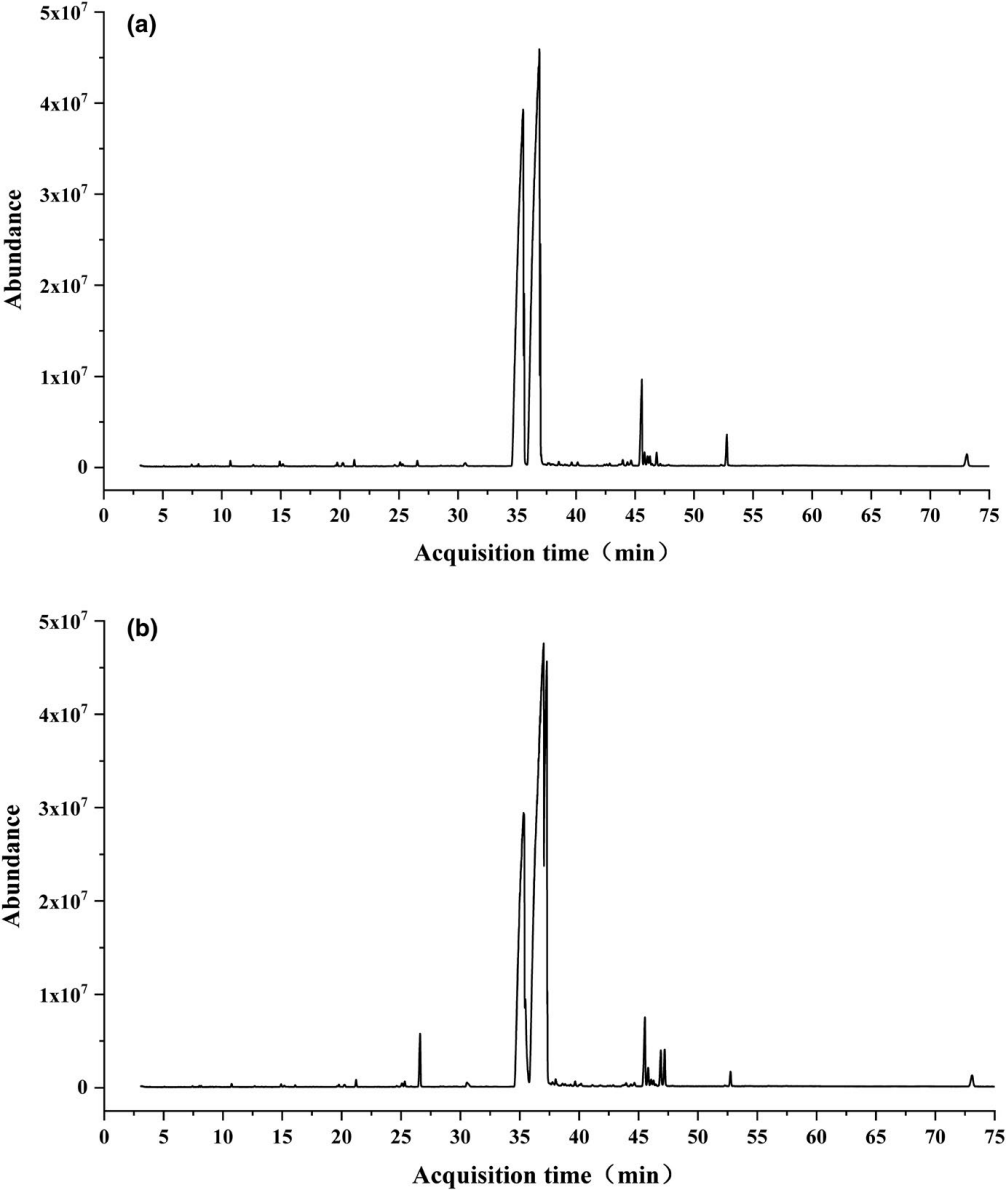
Peak image of purified PNA spectrum detection. The fatty acid composition of pinolenic acid with high purity extracted from Korean pine seed oil was determined by GC‐MS. The results are shown in this figure. (a) The product purified by traditional urea embedding method. (b) Purified product by lipase‐catalyzed esterification combined with urea embedding method

According to the previous experimental results of the research group, high purity pinolenic acid has been extracted from Korean pine seed oil through esterification embedding combined with secondary urea embedding method, and its fatty acid composition has been determined by GC‐MS (Zhou, 2019). The GC‐MS diagram is shown in Figure 1, and the component analysis results are shown in Table 1. Due to the poor solubility of pinolenic acid in culture medium, and considering that DMSO (dimethyl sulfoxide) has certain toxic effects on cells, ethanol was used to dissolve pinolenic acid first. In this test, the final concentration of ethanol in the mother liquor of the prepared sample should not exceed 2%. 9 µl pinolenic acid was added to 291 µl ethanol to prepare the sample mother liquor, which was mixed by vortex oscillation, filtered by 0.22 µm organic filter membrane to remove bacteria, and stored at −20°C.

**TABLE 1:** Main of the fatty acid content of the purified products

Compound name	Korean pine seed oil (%)	Esterification embedding (%)	Urea embedding (%)
Stearic acid	2.03	<1	<1
Palmitic acid	7.16	<1	<1
Oleic acid	27.35	<1	<1
Linoleic acid	46.24	45.47	5.22
Pinotolic acid	17.08	51.42	90.34

**FIGURE 2:**
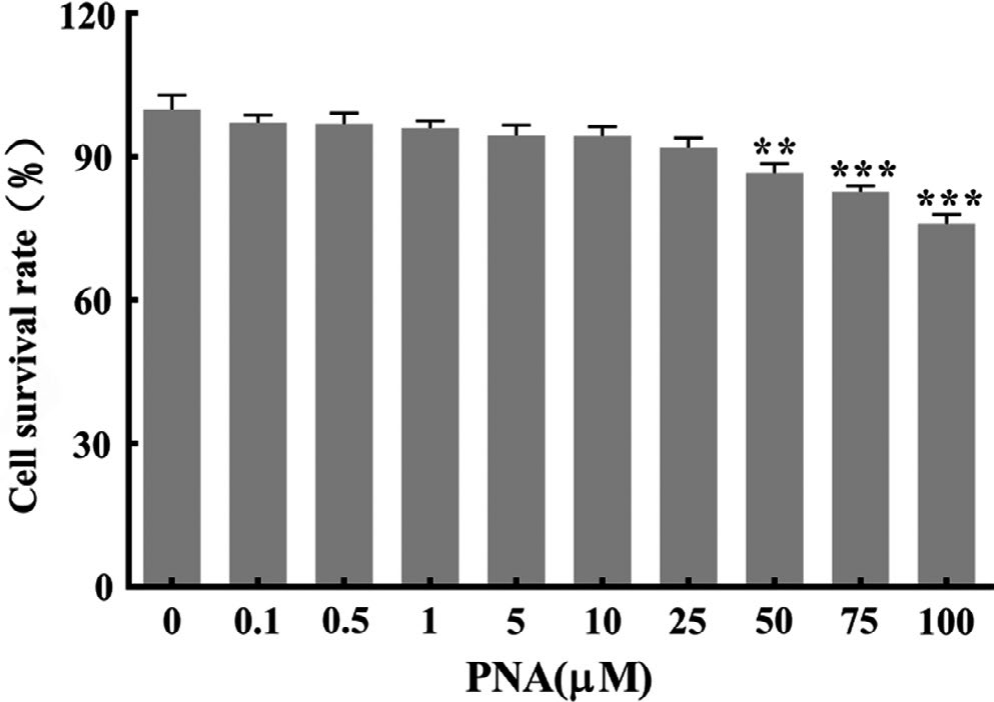
Effect of different concentrations of PNA on the viability of HepG2 cells. Data were expressed as $\bar{x}\pm \text{SD}$. Compared with Mod, **p < .01, ***p < .001

H_2_O_2_ was used to induce oxidative stress of cells to establish the model group. Since the effect of PNA on cell viability is not clear, it may affect the establishment of the model group. Therefore, according to the experimental results of Lee and Han (2016) the effect of pinolenic acid (0.1–100 μM) on survival rate of HepG2 cells was detected. As can be seen from Figure 2, when the concentration of pinolenic acid was 0.1–25 μM, there was no significant effect on cell survival rate. However, when the concentration was 50–100 μM, cell survival rate was significantly affected. Therefore, pinolenic acid with a concentration range of 0.1–25 μM was selected for subsequent tests, to ensure that reduced cell viability in the model group was completely caused by H_2_O_2_.

#### 2.3.3 Cell viability assay

HepG2 cell viability was determined using MTT assay according to Ali Chiroma et al. (2020) with slight modifications. Briefly, HepG2 cells were inoculated at a density of 1 × 10^5^ cells/ml per well in a 96‐well plate. After 24 h, the growth medium was removed and 10 µl of MTT (5 mg/ml) was added to each well and incubated with the cells for 4 hr at 37°C in a 5% CO_2_ atmosphere. Subsequently, 200 µl of DMSO was added to each well with shaking for 10 min at room temperature to dissolve the formazan crystals. The absorbance value was measured using an enzyme‐linked immunosorbent assay (ELISA) reader (BIO‐TEK EL×800, USA) at 570 nm. Results were expressed as percentages of cell viability according to the formula below:

$$\text{Cell viability}\;\left(\%\right)=\left[\frac{\text{(absorbance values of samples)}}{\left(\text{absorbance value of control}\right)}\right]\times 100\%$$

#### 2.3.4 Determination of intracellular ROS production

First, HepG2 cells (1.0 × 10^5^ cells/ml) were seeded in 12‐well plates and treated as designed. The plate containing the cells was incubated in a humidified incubator at 37°C with 5% CO_2_ until further usage. After discarding the culture medium, each well was filled with DCFH‐DA fluorescent probe (10 µM). After incubating at 37°C in the dark for 40 min, cells were washed twice using cool PBS before digesting the cells with trypsin. After centrifuging at 1000 rpm for 8 min, cells were resuspended in PBS, and transferred to the 96‐well plate. Intracellular ROS production was measured by the reactive oxygen species assay kit (Beyotime, China) using a fluorescence microplate reader at the excitation and emission wavelengths of 502 and 530 nm.

#### 2.3.5 Determination of intracellular MDA content

First, HepG2 cells (1.0 × 10^5^ cells/ml) were seeded in 12‐well plates and treated as designed. After 24 h of incubation, cells were collected and centrifuged at 3000 rpm for 10 min. Subsequently, the supernatant was removed and 300 µl of 10% medium followed by sonication in ice for 1 min. After removing the culture solution, 100 µl of pre‐cooled PBS buffer was added to each well, and the cells were subjected to ultrasound. Cells were collected and stored at −20°C. Protein concentration was measured by an enhanced BCA protein assay kit (Biotechnology, China). All operations were performed using the Lipid Peroxidation MDA assay kit according to manufacturer's instructions. MDA content was expressed as mmol/mg protein.

#### 2.3.6 Determination of intracellular antioxidant enzyme levels

SOD, CAT, and GSH‐Px levels were determined according to manufacturer's instructions of the ELISA kits supplied by Jiancheng Bioengineering Institute (Nanjing, China), respectively. The SOD, CAT, and GSH‐Px enzyme activities were expressed as U/mg protein.

#### 2.3.7 Total RNA isolation and qRT‐PCR

Total RNA was extracted, and real‐time polymerase chain reaction (RT‐PCR) was performed with an ABI QuantStudio 6 Flex system (ABI, Carlsbad, CA, USA) according to the manufacturer's instructions. The isolated RNA was then reverse transcribed to cDNA by employing RT‐PCR. The primers sequences for Keap1, Nrf2, HO‐1, and NQO1 genes are shown in Table 2. Forward and reverse primer sequences of the genes of interest were designed and synthesized by Jilin Kumei Biological Technology Co., Ltd. The first‐strand cDNA template of each gene was synthesized using PrimeScript™ RT Reagent Kit with gDNA Eraser (Takara Biotechnology, Japan). The reaction system was TB Green™ Premix Ex TaqTM kit (TaKaRa, DRR420A) where PCR conditions for these primers were 95°C for 30 s, 95°C for 10 s, 57°C for 30 s, and 72°C for 30 s over 40 cycles. All the collected quantitative data were averaged based on quantification cycle (*C*_q_) values, which were used to calculate the fold expression ratio. β‐actin was included as the housekeeping gene for normalization of the target genes.

**TABLE 2:** Primers used in Quantitative Polymerase Chain Reaction

Sl. No.	Gene	Primer
1	*Keap1*	Forward: 5′‐GGCCCTCTCTAGTTCCCAG‐3′ Reverse: 5′‐CAGCATAGATACAGTTGTGCAG‐3′
2	*Nrf2*	Forward: 5′‐GCGTGTAGCCGATTACCGAGTG‐3′ Reverse: 5′‐CATGATGAGCTGTGGACCGTGTG‐3′
3	*HO‐1*	Forward: 5′‐CCTCCCTGTACCACATCTATGT‐3′ Reverse: 5′‐GCTCTTCTGGGAAGTAGACAG‐3′
4	*NQO1*	Forward: 5′‐CCACCTCCTGAGTTCAAGCGATTC‐3′ Reverse: 5′‐GAGTTCAAGACCAGCCTGACCAAC‐3′
5	*β‐actin*	Forward: 5′‐TGGCACCCAGCACAATGAA‐3′ Reverse: 5′‐CTAAGTCATAGTCCGCCTAGAAGCA‐3′

#### 2.3.8 Statistical analysis

Data were expressed as mean ± SD carried out at least in three independent replicates. One‐way analysis of variance (ANOVA) followed by Bonferroni's post hoc test was used to compare the experimental means. Data were analyzed using GraphPad Prism (version 7.0) with model of program. *p* < .05 was considered statistically significant.

## 3 RESULTS AND DISCUSSION

### 3.1 The Effect of PNA on the survival rate of HepG2 cells induced by H_2_O_2_

**FIGURE 3:**
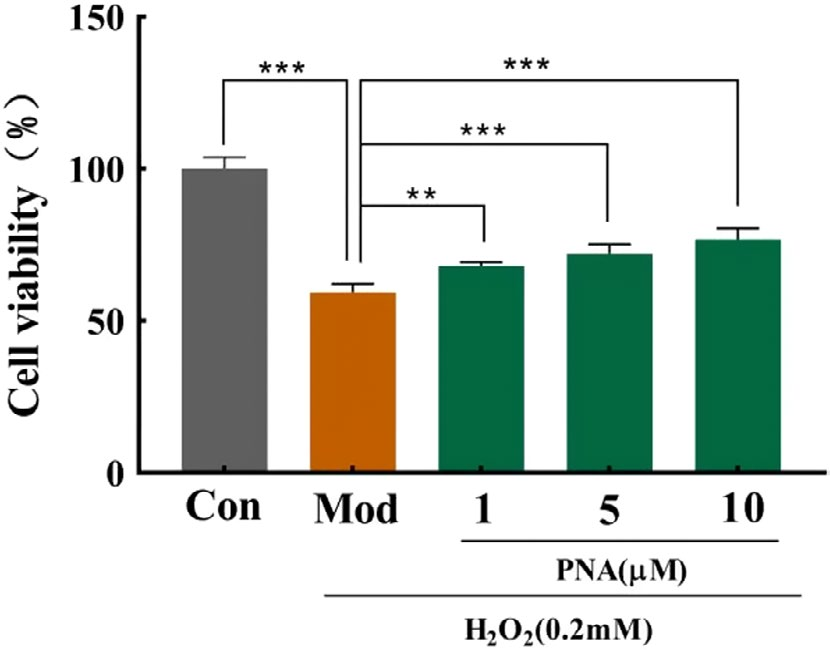
Effect of PNA on HepG2 cells viability induced by H_2_O_2_. HepG2 cells were induced by H_2_O_2_ and regulated by PNA, and then the cell viability was measured. The data were expressed in $\bar{x}\pm \text{SD}$. Compared with Mod, **p < .01, ***p < .001. Excel 2010 was used for preliminary processing of the data

HepG2 cell line is commonly used in cytotoxicity and anti‐proliferative activity assays for their liver‐specific suppression responses toward phytochemicals. Hydrogen peroxide (H_2_O_2_) acts as a cell signaling molecule under normal physiological conditions. However, excessive H_2_O_2_‐induced toxicity in HepG2 cells eventually leads to oxidative stress (Han et al., 2018). Excessive accumulation of ROS causes an increase in intracellular free Ca^2+^ which, in turn, leads to mitochondrial dysfunction, irreversible membrane damage and, finally, cell death (Zhang et al., 2018). Cell viability is the most intuitive indicator to reflect degree of cell damage in order to establish the oxidative stress model. Figure 3 demonstrated that the cell viability of the model group induced by 0.2 mM H_2_O_2_ decreased by 40.7% (*p* < .001) compared with the control group. This indicates obvious cytotoxicity, and hence, the oxidative stress model was established successfully. The cytotoxicity of H_2_O_2_ was dose dependent where cell survival decreased as the concentration of H_2_O_2_ was increased (Figure 3). Supplementation with different doses of PNA (1, 5, and 10 µM) increased the cell survival rates by 14.73, 21.46, and 29.59%, respectively (*p* < .001) compared with the model group.

### 3.2 The effect of PNA on the intracellular ROS content of HepG2 induced by H_2_O_2_

**FIGURE 4:**
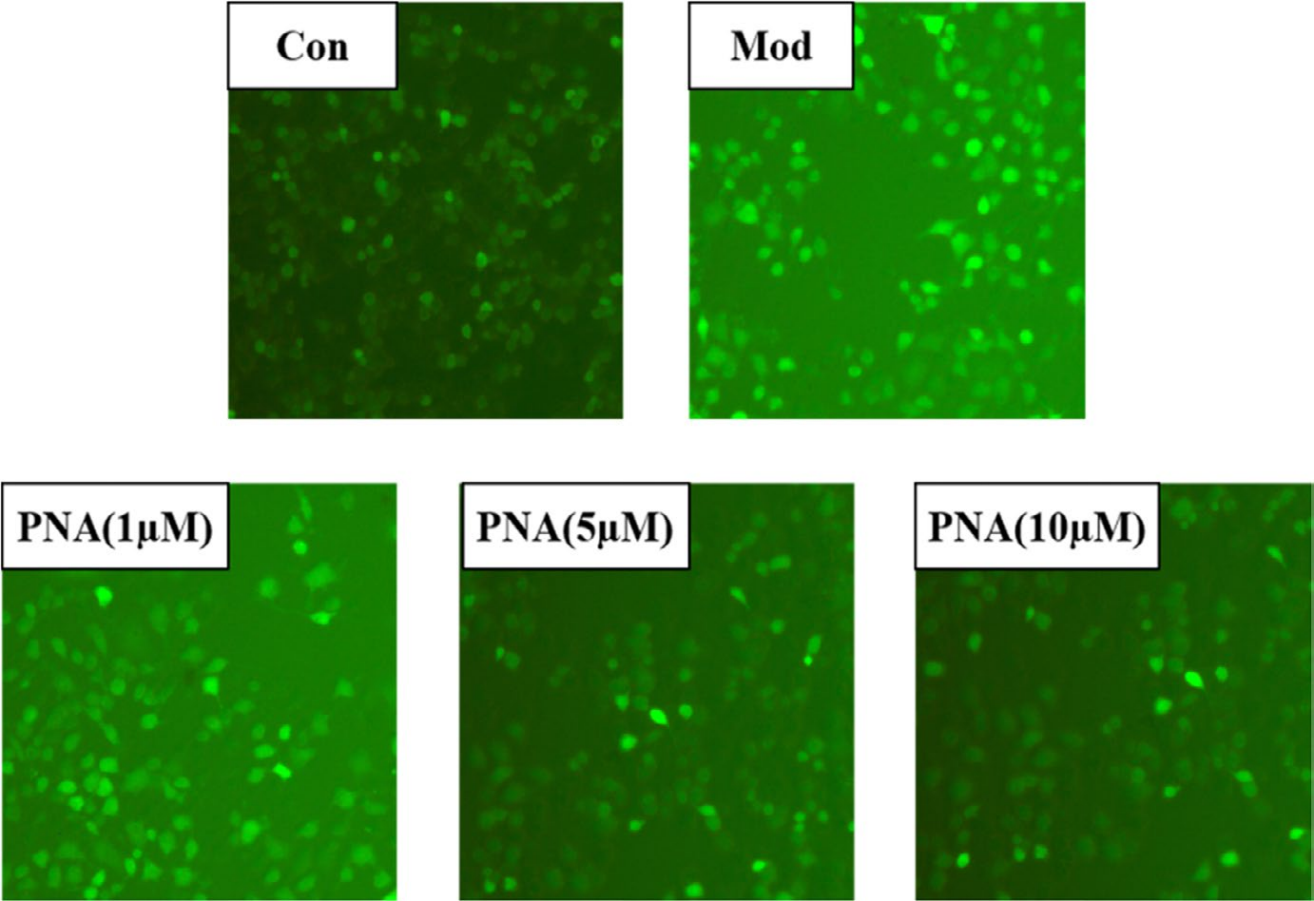
The effect of PNA on ROS activities induced by H_2_O_2_ in HepG2 cells. When ROS is produced, DCFH will be oxidized into DCF (fluorescein) that cannot penetrate the cell membrane. DCF is a strong green fluorescent substance. Therefore, we detected the fluorescence intensity of DCF to reflect the ROS accumulation in the cell

**FIGURE 5:**
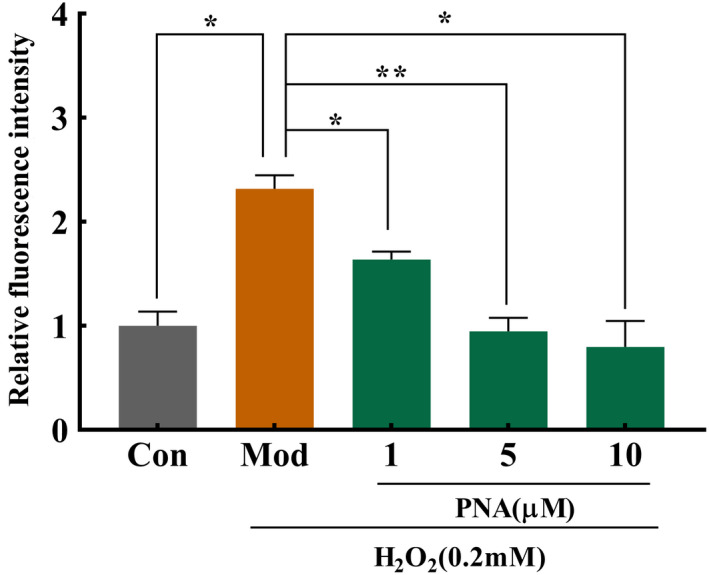
Effect of PNA on ROS activities induced by H_2_O_2_ in HepG2 cells. HepG2 cells were induced by H_2_O_2_ and regulated by PNA, and then the intracellular ROS accumulation was determined according to the relative fluorescence intensity. The data were expressed in $\bar{x}\pm \text{SD}$. Compared with Mod, *p < .05, **p < .01

Exogenous H_2_O_2_ penetrates the cell membrane easily and generates lots of free radicals which attacks the mitochondrial membrane, leading to excessive ROS production in the cells. The changes of intracellular ROS fluorescence are shown in Figure 4. Fluorescence images showed in Figure 4 suggested that the model group had the highest ROS production indicating cell damage compared with the control group. Supplementation with PNA decreased the fluorescence intensity effectively (Figure 4). Figure 5 showed that the effect of PNA on the intracellular ROS content of HepG2 measured according to relative fluorescence intensity. The relative fluorescence intensity of intracellular ROS increased significantly (*p* < .05) by 132% after induced by H_2_O_2_ compared with the control group. The relative fluorescence intensity of intracellular ROS decreased significantly (*p* < .05) by 29.1, 59.1, and 65.5%, respectively after 1, 5, and 10 µM PNA supplementation. Cells treated with high‐dose PNA (10 µM) had the lowest accumulation of intracellular ROS (*p* < .05) where this was even lower than the control group (Figure 5). These results suggested that PNA intervention decreased ROS production effectively caused by H_2_O_2_.

### 3.3 The effect of PNA on the MDA level of HepG2 induced by H_2_O_2_

**FIGURE 6:**
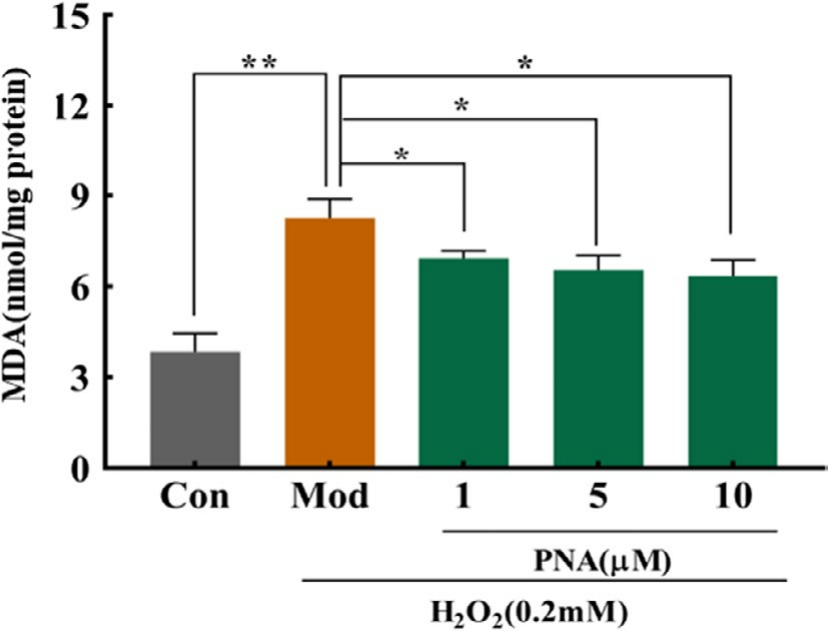
Effect of PNA on MDA activities induced by H_2_O_2_ in HepG2 cells. HepG2 cells were induced by H_2_O_2_ and regulated by PNA, and then intracellular MDA content was detected. The data were expressed in $\bar{x}\pm \text{SD}$. Compared with Mod, *p < .05, **p < .01

MDA is the standard biomarker commonly used to measure lipid peroxidation of cell membrane despite other biomarkers such as 4‐hydroxynonenal and isoprostanes. MDA is generated from the peroxidation of polyunsaturated fatty acids with more than three double bonds where it is the most abundant active carbonyl (Musa et al., 2017). It can be seen from Figure 6 that the MDA content of HepG2 cells in the model group increased significantly by 114.45% (*p* < .01) compared with the control group due to 24 h incubation with H_2_O_2_. Intracellular MDA content decreased significantly (*p* < .05) by 15.96%, 20.5%, and 22.9%, respectively after 1, 5 and 10 µM treatment using PNA (Figure 6). Results indicate that PNA resisted lipid peroxidation caused by ROS, relieving cellular oxidative damage caused by free radicals.

### 3.4 The effect of PNA on the antioxidant enzyme system of HepG2 induced by H_2_O_2_

**FIGURE 7:**
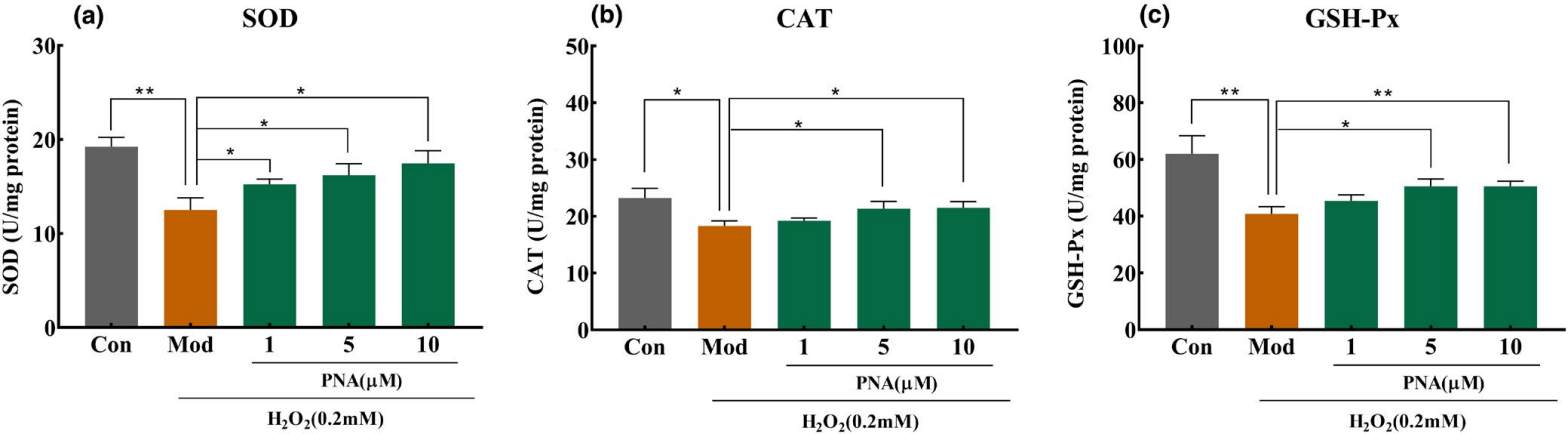
Effect of PNA on antioxidant enzyme induced by H_2_O_2_ in HepG2 cells. HepG2 cells were induced by H_2_O_2_ and regulated by PNA, and then the activity of SOD (a), CAT (b), and GSH‐Px (c) was detected. The data were expressed in $\bar{x}\pm \text{SD}$. Compared with Mod, *p < .05, **p < .01

SOD, CAT, and GSH‐Px are important components of the cellular antioxidant enzyme system. It is important as the antioxidant defense system to maintain the dynamic balance of oxidative stress (Niki, 2010). Under oxidative stress, these antioxidant enzymes will act as endogenous antioxidant to scavenge free radicals intracellularly (Musa et al., 2017). SOD catalyzes the decomposition of superoxide anion into H_2_O_2_ and O_2_, which in turn scavenge free radicals. CAT and GSH‐Px catalyze the decomposition of H_2_O_2_ into H_2_O to maintain redox balance of the organism by eliminating excessive free radicals produced in vivo. It can be seen from Figure 7 that the enzymatic activities of SOD, CAT, and GSH‐Px in HepG2 cells reduced significantly (*p* < .05) by 35.04%, 21.15%, and 34.17%, respectively, compared with the control group after 24 hr incubation with H_2_O_2_. However, the enzymatic activity of SOD increased significantly (*p* < .05) by 21.8%, 29.6%, and 39.7%, respectively, after treatment using 1, 5, and 10 µM PNA (Figure 7a). The enzymatic activity of CAT increased by 4.95%, 16.6%, and 17.6%, respectively, while the enzymatic activity of GSH‐Px increased by 11.1%, 23.6%, and 23.8%, respectively, after treatment using 1, 5, and 10 µM PNA (Figure 7b,c). Medium and high dosages of PNA (5 and 10 µM) increased the enzymatic activities of CAT and GSH‐Px significantly (*p* < .05) while 1 µM PNA had no significant effect (*p* > .05). These results indicated that treatment with PNA improved the endogenous antioxidant defense.

### 3.5 The effect of PNA on the antioxidant genes of HepG2 induced by H_2_O_2_

**FIGURE 8:**
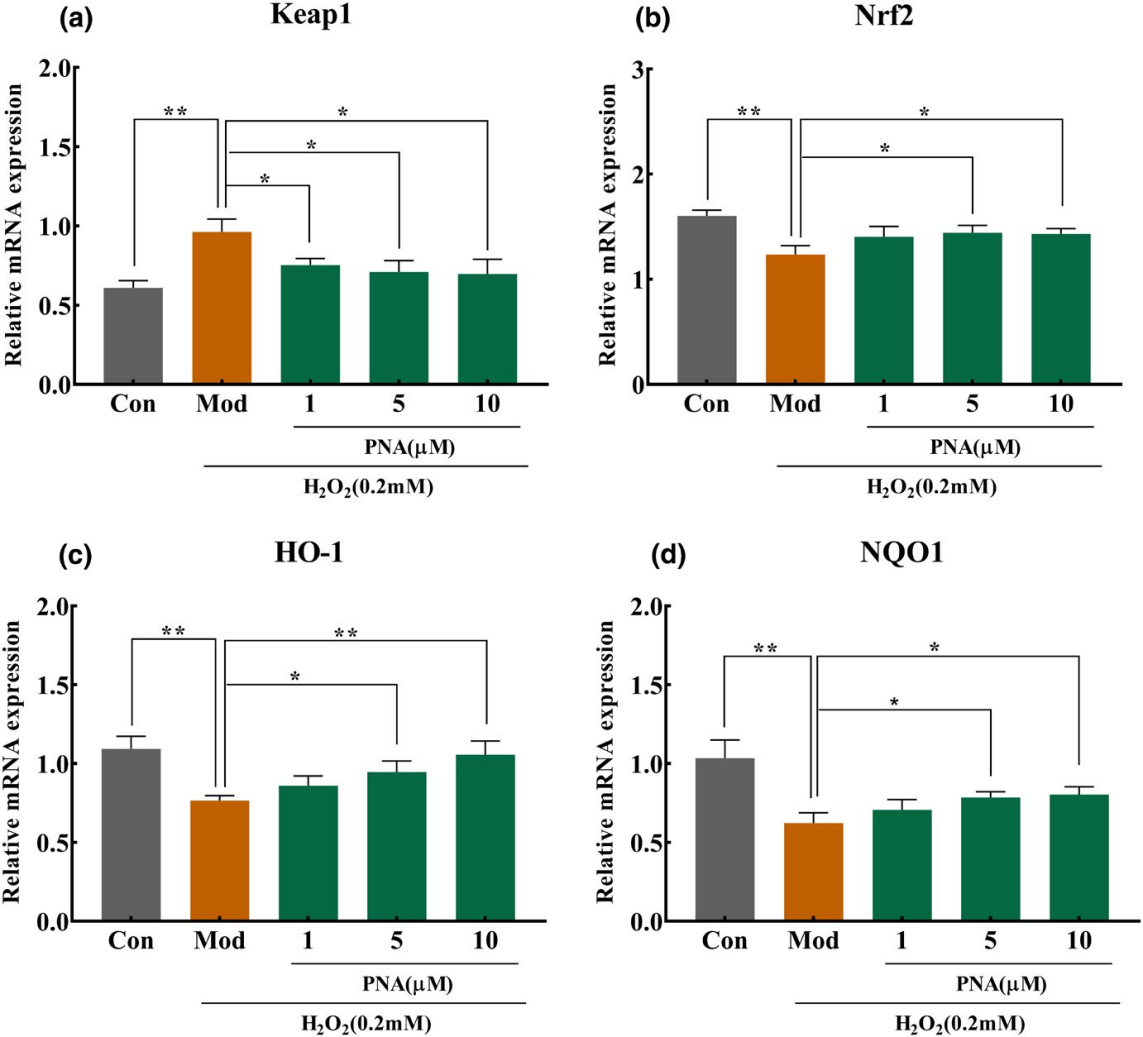
Effect of PNA on mRNA expression of antioxidant genes in HepG2 cells. HepG2 cells were induced by H_2_O_2_ and regulated by PNA, and then the mRNA expression of Keap1 (a), Nrf2 (b), HO‐1 (c), and NQO1 (d) was detected. The data were expressed in $\bar{x}\pm \text{SD}$. Compared with Mod, *p < .05, **p < .01

Antioxidant enzymes were used by cells to resist excess ROS to maintain redox balance. When the redox maintains a dynamic balance in cells, Nrf2 will be combined with Keap1 in the cytoplasm and maintain a static state. Once oxidative stress damage occurs in the cell, Nrf2 will dissociate from Keap1, enter the nucleus and interact with ARE, and then up‐regulate antioxidant enzyme genes downstream of Nrf2, such as HO‐1 and NQO1, to achieve the purpose of protecting cells from oxidative stress. Figure 8a demonstrated that the expression level of Keap1 gene in the model group increased significantly (*p* < .01) by 57.9%. 1, 5, and 10 µM PNA reduced the expression level of Keap1 gene significantly (*p* <.05) by 21.8%, 26.3%, and 27.62%, respectively, compared with the control group. The Nrf2 transcription factor is the master regulator in cellular antioxidant defense responses by activating ARE phase II antioxidant enzymes, such as HO‐1 (Lever et al., 2016). Figure 8b demonstrated that the expression level of the Nrf2 gene of the model group decreased significantly (*p* < .01) by 22.8% compared with the control group. After treatment with 1, 5, and 10 µM PNA, the expression level of Nrf2 gene in HepG2 cells increased by 13.7%, 16.7%, and 15.8%, respectively, compared with the control group. However, the expression level of Nrf2 increased significantly (*p* < .05) only in cells treated with medium‐ and high‐dose groups (5 and 10 µM).

HO‐1 is known as a stimulation response protein that is induced in various stress conditions. HO‐1 is one of the most representative ARE response enzyme regulated by Nrf2 (Loboda et al., 2016). As shown in Figure 8c,d, the expression levels of NQO1 and HO‐1 genes reduced significantly (*p* < .01) by 29.8% and 39.9%, respectively, compared with the control group. However, treatment with 1, 5, and 10 µM PNA increased the expression level of HO‐1 gene by 12.1%, 23.5%, and 37.8%, respectively, compared with the control group. In relation, treatment with 1, 5, and 10 µM PNA also increased the expression level of NQO1 gene by 13.5, 26.3, and 28.9, respectively. However, cells treated with medium and high doses of PNA (5 and 10 µM) demonstrated significant increase (*p* < .05) of the expression levels of HO‐1 and NQO1 genes compared with the model group. It was well‐known that down‐regulation of Nrf2 expression level affected the cellular antioxidant defense responses, resulting in high ROS production that caused cell death (Yu et al., 2019). When HepG2 cells were treated with PNA, the expression levels of Nrf2, HO‐1, and NQO1 increased at a dose‐dependent manner. This event further activates the production of antioxidant enzymes, such as SOD, CAT, and GSH‐Px. Hence, MDA production was decreased. All these events enhanced the antioxidant defense mechanisms, increasing the cellular antioxidant capacity to alleviate high ROS production.

## 4 CONCLUSION

The experimental results showed that the cell survival rate was significantly improved when PNA was used to modulate oxidative stress injury induced by H_2_O_2_ in HepG2 cells, which proved that PNA has the function of alleviating cellular oxidative stress and improving cellular oxidative stress injury. The results of the ROS accumulation showed that PNA reduced intracellular ROS levels, which further indicated that the intensity of oxidative stress in HepG2 cells induced by H_2_O_2_ was weakened. From the results of the reduction of intracellular MDA content, PNA not only can weaken the oxidative stress response, but also improve the damage caused by oxidative stress;

Besides, the activity of SOD, CAT, and GSH‐Px was enhanced under the regulation of PNA, which preliminarily revealed the pathway that PNA regulated oxidative stress—the intervention from PNA can increase the content of cellular antioxidant enzymes. PNA can alleviate the damage caused by free radicals to cellular antioxidant enzymes; therefore, it can reduce oxidative stress damage and enhancing the body's antioxidant capacity.

To further reveal the antioxidant pathway of PNA, this experiment used qRT‐PCR to detect mRNA expression of related antioxidant genes in HepG2 cells under the regulation of PNA. The results showed that PNA can dissociate the compound by down‐regulating the expression level of Keap1 gene and up‐regulating the expression level of Nrf2 gene; therefore, the expression levels of the antioxidant enzyme genes HO‐1 and NQO1 genes in the downstream of Nrf2 were improved, so as to improve the activity of antioxidant enzymes and cellular oxidative stress.

## CONFLICT OF INTEREST

None of the authors have conflict of interests regarding this research.

## AUTHOR CONTRIBUTIONS

**Yang Zhao:** Data curation (lead); Formal analysis (lead); Writing‐original draft (equal); Writing‐review & editing (equal). **Sainan Liu:** Data curation (supporting); Formal analysis (supporting); Investigation (supporting); Writing‐original draft (supporting); Writing‐review & editing (supporting). **Zhili Sheng:** Conceptualization (equal); Data curation (supporting); Writing‐original draft (supporting). **Xue Li:** Conceptualization (supporting); Investigation (supporting). **Yanan Chang:** Data curation (supporting); Investigation (supporting). **Weichang Dai:** Formal analysis (supporting); Methodology (supporting). **Sui Kiat Chang:** Conceptualization (supporting); Formal analysis (supporting). **Junmei Liu:** Funding acquisition (equal); Investigation (equal); Methodology (equal); Project administration (equal); Resources (equal); Writing‐review & editing (equal). **Yuchun Yang:** Funding acquisition (equal); Methodology (equal); Project administration (equal); Writing‐review & editing (equal).

## Data Availability

Research data are not shared.
